# NREM Sleep Parasomnias Commencing in Childhood: Trauma and Atopy as Perpetuating Factors

**DOI:** 10.3390/clockssleep4040043

**Published:** 2022-10-17

**Authors:** Cara Walsh, Lee Mitchell, Maria Hrozanova, Serafeim-Chrysovalantis Kotoulas, Christopher Derry, Ian Morrison, Renata L. Riha

**Affiliations:** 1 Sleep Research Unit, Department of Sleep Medicine, The University of Edinburgh, Royal Infirmary of Edinburgh, 51 Little France Crescent, Edinburgh EH16 4SA, Scotland, UK; 2 Department of Neurology, Ninewells Hospital, Dundee DD1 9SY, Scotland, UK

**Keywords:** slow-wave sleep parasomnia, childhood, adolescence, adulthood, trauma, arousal disorder, atopy

## Abstract

Objective/Background: Phenotyping of non-rapid-eye-movement (NREM) parasomnias is currently poorly undertaken. This study aimed to determine whether there are differences phenotypically among childhood-, adolescent-, and adult-onset NREM parasomnias continuing into and presenting in adulthood. Patients/Methods: A retrospective, cohort study of patients presenting with NREM parasomnia between 2008 and 2019 (*n* = 307) was conducted. Disorders included sleepwalking (*n* = 231), night terrors (*n* = 150), sexualised behaviour in sleep (*n* = 50), and sleep-related eating disorder (*n* = 28). Results: Compared to the adult-onset NREM behaviours group, the childhood- and adolescent-onset groups were more likely to have a family history of NREM behaviours (*p* < 0.001), experience a greater spectrum of NREM disorders (*p* = 0.001), and report a history of sleep-talking significantly more frequently (*p* = 0.014). Atopy was most prevalent in the childhood-onset group (*p* = 0.001). Those with childhood-onset NREM parasomnias were significantly more likely to arouse from N3 sleep on video polysomnography (*p* = 0.0003). Psychiatric disorders were more likely to be comorbid in the adult-onset group (*p* = 0.012). A history of trauma coinciding with onset of NREM behaviours was significantly more common in the childhood- and adolescent-onset groups (*p* < 0.001). Conclusions: Significant differences exist across childhood-, adolescent-, and adult-onset NREM parasomnia presenting in adulthood. This study suggests that adult-onset slow-wave sleep disorders may be confounded by psychiatric disorders resulting in nocturnal sleep disruption and that unresolved traumatic life experiences perpetuate NREM disorders arising in childhood and comprise one of the strongest external risk factors for triggering and perpetuating these disorders in adolescence.

## 1. Introduction

Non-rapid-eye-movement sleep (NREM) parasomnias occur throughout the lifespan [1]. According to the International Classification of Sleep Disorders (third edition, ICSD-3), they include sleepwalking, sleep-related eating disorder (SRED), night terrors, and sexualised behaviour in sleep (SBS) [2]. Sleep-talking is no longer included under NREM parasomnias in ICSD-3 [2].

Sleepwalking appears to be more prevalent in childhood compared to adults (14.5% vs. 1.7%) [3,4], whereas SRED and SBS almost exclusively affect adult patients. Sleepwalking has been associated with a specific human leukocyte antigen (HLA) isotype (HLA-DQB1*05:01), associated with asthma and pollen allergy, but this link has not been established to date [5]. It is unknown whether this HLA is more common in childhood- or adult-onset NREM parasomnia. Paediatric NREM parasomnias are common and generally do not exhibit complex or dangerous behaviours; they are usually self-limiting and resolve in adolescence, but sometimes can continue into adulthood [6]. Individuals with childhood NREM parasomnia are also more likely to have a positive family history compared to their adult counterparts [7].

Less commonly, NREM parasomnia may first appear in adulthood [6]. NREM parasomnia in adulthood can appear to involve executive function and more violence than in childhood and can have serious medico-legal consequences [7].

Presentation of de novo NREM parasomnia in adults has been associated with the presence of psychopathology [8]. Sleepwalking is more prevalent in patients with a psychiatric comorbidity compared to those without (8.5% vs. 2.0%), and patients with a comorbid eating disorder are more likely to have SRED compared to the general population (16.7% vs. 4.6%) [9]. Adult patients in a psychiatric population exhibit more frequent and complex episodes of NREM parasomnia compared to those in a childhood-onset population [9]. Dissociation is a differential diagnosis for NREM parasomnia in association with psychiatric comorbidity. However, nocturnal dissociation is no longer recognised as a sleep disorder and is not included in ICSD-3 [2].

Lifestyle changes such as sleep hygiene advice, with a focus on caffeine reduction, exercise, and a decrease in using blue-light-emitting screens close to bedtime, are first-line interventions in children and have been found to alleviate NREM parasomnias in 12.9% of patients [1,10], with pharmaceutical management deemed a last resort [11]. Pharmacological therapy is often the first-line treatment for adults who exhibit frequent, complex, or harmful behaviour arising out of sleep [10]. Clonazepam, a benzodiazepine, is the most frequently prescribed drug, reported to alleviate symptoms in 72.7% of patients in one cohort [12]. Overall success rates for antidepressants such as citalopram and mirtazepine, non-benzodiazepine sedatives such as zolpidem, and melatonin were found to range between 58% and 77% [12]. Psychological therapy such as counselling, cognitive behavioural therapy (CBT), and hypnotherapy may be indicated for the treatment of NREM parasomnias if the individual experiences adverse drug effects or has recognised and associated emotional triggers [1]. All of these interventions have been identified as partially successful in symptom control in NREM parasomnias [13,14,15].

More recent studies have started to explore some of the differences and changes over the lifespan in relation to the expression of NREM parasomnias. For example, in 165 adult sleepwalkers, Kalantari et al. [16] reported that the episode frequency of sleepwalking increased over the lifespan from childhood, as did dream mentation recall associated with the sleepwalking. They also reported that aggression became more frequent in adulthood. These findings were mirrored in a study by Castelnovo et al. [17], which demonstrated reduced dream mentation activity during NREM episodes in children compared to adults. Lastly, studies exploring genetic associations of NREM parasomnias are increasing in number, with the study by Chiba et al. [18] most recently identifying a genetic risk locus in one ethnic group but not another in a large paediatric sleepwalking cohort, very much in contrast to previous findings [5,19].

The aim of this study was to investigate the characteristics, comorbidities, and course of disease comparatively in childhood-, adolescent-, and adult-onset NREM parasomnia presenting in adolescents and adults, as well as responses to treatment between the groups. Very few studies have directly compared these age-of-onset groups in SWS disorders. The importance of such work to phenotyping, cannot be underestimated.

## 2. Results

A total of 307 patients were included in the analysis, of whom 133 (43%) were male and 174 (57%) were female. The minimum age in the cohort was 14 years, and the maximum age was 76 years. There was no significant difference in the sex ratio among patients with childhood-, adolescent-, and adult-onset parasomnia. Patients with childhood- and adolescent-onset parasomnia were significantly younger at presentation compared to patients with adult-onset parasomnia (31.1 ± 9.9 vs. 29.5 ± 9 vs. 39.5 ± 15.0 years, *p* < 0.001) (see Table 1). There were no significant differences among the three groups in terms of SIMD deprivation score, body mass index (BMI), Epworth sleepiness scale (ESS), subjective hours of sleep per night, smoking, and caffeine consumption. Interestingly, patients with childhood-onset parasomnia seemed to consume alcohol as adults more often (occasionally to regularly) compared to patients with adult-onset parasomnia (*p* = 0.012). There were no significant differences across the groups in terms of marital status, shift work, and self-reported sleep quality. 

As expected, patients with childhood-onset parasomnia had a longer duration of the disorder compared to patients with adult-onset parasomnia (*p* < 0.001) (Table 2). Patients with childhood- and adolescent-onset parasomnia were more likely to have a positive family history of NREM parasomnias and report a greater spectrum of NREM behaviours compared to patients with adult-onset parasomnia (35.9% vs. 48% vs. 21.4%, *p* = 0.005 and 82.6% vs. 96% vs. 67.8%, *p* = 0.001, respectively) (Table 2). There were no differences across the groups in the frequency of the behaviours. However, conscious awareness for the behaviour was least likely to be reported by the adolescent-onset group with partial amnesia being very common compared to the other two groups (*p* < 0.001) (Table 2).

Sleepwalking and night terrors were significantly more common in patients with childhood- and adolescent-onset parasomnia compared to those with adult-onset parasomnia (*p* < 0.001), whereas SBS was most common in patients with adult-onset parasomnia (*p* = 0.005) (Table 3). There was no significant difference among the three groups in the prevalence of SRED (Table 3). Patients with childhood- and adolescent-onset parasomnia were also more likely to present with other sleep disturbances such as sleep-talking and more frequent dream mentation, compared to patients with adult-onset parasomnia (*p* = 0.014 and *p* < 0.001, respectively) (Table 3). On the other hand, there were no differences between the two groups in the frequency of violence in sleep and in the prevalence of other sleep disorders such as self-reported insomnia, bruxism, sleep paralysis, obstructive sleep apnoea (OSA), and restless legs syndrome (RLS) (Table 3). Overall, a longer duration of the NREM parasomnia was correlated with a greater frequency of behaviours (*r* = 0.126, *p* = 0.045) and recollection of dream mentation (*r* = 0.124, *p* = 0.035).

On vPSG, patients with childhood- and adolescent-onset parasomnia had a lower apnoea–hypopnoea index (AHI) compared to patients with adult-onset parasomnia (*p* = 0.005); none of the other PSG variables reached statistically significant differences across the three groups (see Table 4). However, those with childhood onset NREM parasomnias were significantly more likely to arouse out of N3 sleep with a behaviour on vPSG, compared to those with adolescent- and adult-onset parasomnias (64% vs. 16% vs. 48%, *p* = 0.0003). 

The frequency of asthma was not significantly different across all three groups (13.5% vs. 20% vs. 13.5%, *p* = 0.61), whereas patients with childhood-onset NREM parasomnia had a significantly higher prevalence of atopy compared to patients with adolescent- and adult-onset parasomnia (16.7% vs. 0% vs. 4.8% respectively; *p* = 0.001). Patients with asthma were significantly more likely to be sleepwalkers compared to non-asthmatics (*n* = 31/43; *p* < 0.0001). Patients with atopy were more likely to have a history of sleep-talking (*n* = 23/32; *p* = 0.005) and less likely to report depression in association with their NREM parasomnia (*n* = 25/32; *p* = 0.022). 

Patients with childhood-onset parasomnia had a significantly lower prevalence of psychiatric comorbidities compared to those with adolescent- and adult-onset parasomnia (37.2% vs. 48% vs. 54.8%, *p* = 0.012) (Table 5). Patients with childhood- and adolescent-onset parasomnia were more likely to have experienced trauma around the time of onset of their disorder compared to patients with adult-onset parasomnia (78.7% vs. 100% vs. 40.5%, *p* < 0.001) (Table 6).

Regarding therapy prescribed, there were no differences across the groups (Table 7) although pharmacological treatments were most frequently trialled in all patient groups (>70%). Patients with childhood-onset parasomnia were most likely to be prescribed amitriptyline or clonazepam, with clonazepam also being trialled in the majority of patients (statistical significance not achieved) (Table 8).

## 3. Discussion

This study is the first to compare the treatment response and course of disease across childhood-, adolescent-, and adult-onset NREM parasomnias only presenting in adolescence and adulthood, with the largest sample size of any study to date.

Our results show that sleepwalking and night terrors are more likely to begin in childhood than in adulthood, whereas the opposite applies to SBS. This is in accordance with previous literature on NREM parasomnias [3,4]. In a study investigating NREM parasomnias in a psychiatric population, the adult-onset group was more likely to suffer from comorbid SRED [9]. This finding was not replicated in our study, which may represent a more general population presenting to tertiary sleep disorder clinics rather than a psychiatric subpopulation of patients. 

A positive family history for NREM disorders was significantly more common in patients with childhood- and adolescent-onset NREM parasomnias, as documented in previous studies [7]. This difference may reflect a difference in the aetiology and pathophysiology between the groups; childhood parasomnias may result from additional central nervous system (CNS) immaturity, whereas adult-onset parasomnias are linked more strongly to underlying psychiatric illness [6,11]. However, the aetiology overall is likely to be far more complex. 

Multiple NREM behaviours were found to occur significantly more frequently in the childhood- and adolescent-onset groups than the adult-onset group. There is no clear explanation for this finding, which has not been reported in previous studies, but again may be linked to a genetic component which may exert a pleiotropic effect on the behaviours. In the study cohort overall, a longer duration of NREM parasomnia, irrespective of age at onset, was correlated with a greater frequency of the behaviours at night and greater dream mentation recall, a finding which supports the more restricted phenotypes and numbers in recent studies [16,17].

In the past, behaviour arising out of N3 on vPSG was considered diagnostic of NREM parasomnias. However, a study by Fois et al. found that vPSG confirmed the diagnosis in only 60.5% of patients with NREM parasomnia [23]. By contrast, we captured only 37.5% of our population with behaviours arising out of NREM sleep, with a significantly greater number in the childhood-onset group. Although useful, vPSG appears to have low sensitivity as a diagnostic tool for NREM parasomnias. The percentage of N3 was low by contrast to that reported in previous studies for reasons which are unclear. However, we only performed one night of monitoring and did not use a sleep deprivation protocol routinely as may be the case in other centres. There are no normative data on arousal frequency out of N3 sleep in either children or adults in the community at large, although two recent studies [24,25] showed that, on even one night of PSG, those with NREM parasomnias had significantly greater arousal frequency compared to normal controls. 

In our cohort, asthma prevalence was twice that reported in the general Scottish population (14% vs. 7.1%) [26], whereas the prevalence of reported atopy was half that reported in Scotland (10% vs. 20–25%) [27]. However, atopic disease was found to be significantly higher in the childhood-onset group than the adult-onset group. HLA-DQB1*05:01 has been linked to sleepwalking but is also associated with asthma and pollen allergies [5]. This HLA allele could represent a genetic marker for asthma, atopy, and NREM parasomnias. Previously, 35% of individuals in an SWS parasomnia cohort were found to carry this HLA allele, compared to 13.3% of the general population [28]. Additionally, we speculate that asthmatic children may have a fear of breathlessness at night, which could be linked to the development of an NREM disorder, with anxiety and hypervigilance surrounding sleep in general. Similarly, atopy will interfere with sleep at night by inducing breathing difficulties (hay fever) or scratching/discomfort (eczema).

Diagnosed psychiatric disorders were significantly more prevalent in the adult-onset, group suggesting that psychiatric illness may precipitate and propagate NREM disorders. This reflects other publications in the area suggesting that NREM parasomnia is associated with psychiatric disorders, and that adult-onset NREM parasomnia is more prevalent in psychiatric populations [9]. However, there were no significant associations found between any specific psychiatric disorder and age at onset when measured separately, which contrasts with research suggesting that rates of depression and bipolar disorder are significantly higher in adult sleepwalker populations [29].

A history of trauma coinciding with onset of NREM behaviours was significantly more common in the childhood- and adolescent-onset groups compared to the adult-onset group, indicating that trauma may be a precipitating or propagating factor in NREM parasomnias. In our cohort of patients, the prevalence of psychological trauma (32.6%) was much lower than that of the general Scottish population which has been reported to be as high as 51–61% [30]. This may be due to under-reporting and a failure to question the patient directly, a recognised phenomenon in clinical practice [31].

In our cohort, there were no significant associations between age of onset of parasomnia and sex. Similarly, other studies have not reported sex differences within their cohort [4,9]. The higher number of females in this study may be due to sex-specific health-seeking behaviour, whereby women are more likely to seek medical care and show greater concern about health-related matters than men in general [32].

Socioeconomic status (SES) has also been found to influence health-seeking behaviour, with a higher SES indicating a greater interest in seeking health advice [33]. With an average SIMD deprivation score of 4 (least deprived), this cohort may have been biased against individuals who do not consider their NREM disorder an issue or were reluctant to seek further help.

Clonazepam was the most frequently prescribed drug in all groups and reflects the current empirical treatment suggested in the literature [12]. In this study, prescription of clonazepam and amitriptyline was significantly higher in the childhood-onset group. This has not been previously reported. A recent review of treatments for NREM disorders found that there was a limited evidence base for pharmacological treatments in general for management of NREM disorders, specifically due to the lack of adequately powered, randomised controlled trials [34]. Overall success rates of non-benzodiazepines including sertraline as a treatment for NREM parasomnia were found to be 58–77% [12]. The reason for this may be reflected in the greater co-occurrence of psychiatric disorders in adult-onset NREM parasomnias, particularly anxiety and depression. The small numbers of patients referred for talking therapies reflects the dearth of resources in the public system, where, out of frustration and necessity, medication has become the primary tool in the armamentarium.

The largest limitation to this study is the lack of a ‘normal’ control cohort, as well as possible self-presentation bias. To reduce patient recall bias and misreporting, bed partners were interviewed or asked to provide written information prior to the patient’s attendance at clinic. The possible nondisclosure of traumatic experiences by patients, undiagnosed psychiatric disorders, and atypical sleep on the first night of a vPSG (without sleep deprivation) may have produced results that may not have been entirely representative of the clinical problems. Due to possible patient nondisclosure and occasional lack of direct questioning by interviewing physicians, the prevalence of trauma in this cohort may be lower than recorded. We strongly advocate for future work to focus on the link between traumatic life experiences and NREM parasomnias. Lastly, due to the generally benign nature of NREM parasomnias arising in childhood, this study lacked a ‘childhood only’ NREM parasomnia group, i.e., those who experienced NREM parasomnias in childhood without continuation into adulthood. In the future, the inclusion of a such a group may provide key insights into the differences in aetiopathogenesis between childhood- and adult-onset NREM parasomnias and those who continue to experience their parasomnia lifelong. 

## 4. Material and Methods

This retrospective, cohort study included 307 patients sequentially referred to a tertiary sleep medicine centre between 2005 and 2019. Patients were reviewed by a sleep medicine specialist, and video polysomnography (vPSG) was undertaken in 214 (70%) individuals. Each patient was formally diagnosed with one or more NREM parasomnias according to the ICSD-3 classification [2]. The ICSD-3 criteria do not require VPSG for diagnosis although it can be used to provide corroborative evidence in support of the diagnosis [2]. General diagnostic criteria for NREM parasomnias include recurrent episodes of incomplete awakening from sleep, inappropriate or absent responsiveness to efforts of others to intervene or redirect the person during the episode, limited or no associated cognition, partial or complete amnesia for the episode, and episodes unable to be explained by any other sleep disorder, mental disorder, medical condition, medication, or substance use [2]. With respect to self-reporting parasomnias, simple and complex behaviours were characterised according to the criteria of Loddo et al., bearing in mind that these were created in the context of PSG-recorded behaviour [20] Complex behaviours were defined as interaction with the environment, associated with additional behaviours, e.g., speaking, screaming, and semi-purposeful movement [20].

Onset of parasomnia was classified as childhood onset (up to 13 years age), adolescence onset (13–17 years age), and adult onset (18 years of age or older). 

Patient details were extracted from electronic case notes and entered into a database. The database included information on the demographics of the patient population, as well as information from a questionnaire completed prior to admission to the tertiary sleep medicine centre. Video-PSG results were obtained using ProFusion PSG 4 Compumedics™ software (including previous iterations of the software) within the sleep medicine laboratory. Video-PSG data were scored by qualified sleep physiologists in accordance with the American Academy of Sleep Medicine scoring manual guidelines [35]. Studies were undertaken on one night only; the centre does not use sleep deprivation protocols. No patients were taking medication known to suppress NREM sleep, e.g., benzodiazepines. The presence of arousals out of NREM, demonstrating behaviour consistent with a NREM parasomnia, was noted and used to support the diagnosis. Patients with overlap parasomnia were excluded as were any patients with REM sleep without atonia irrespective of cause, e.g., overlap, secondary to medication. Comorbidities and past medical history were recorded. Pharmacological and other treatments for NREM parasomnias were noted.

Where a history of psychological trauma was recorded, it was graded by severity and age at occurrence. Traumatic experience was defined as bearing witness to “a stressful event or situation (of either brief or long duration) of an exceptionally threatening or catastrophic nature, which is likely to cause pervasive distress in almost anyone” [21]. Patients were judged to have a psychiatric disorder on the basis of a diagnosis made by a mental health professional using the Diagnostic and Statistical Manual of Mental Disorders, Fifth Edition (DSM-5) [22]. Psychiatric disorders were included in analyses on the basis of the individual ever being diagnosed. Individuals were assigned a deprivation score according to their postcode using the Scottish Index of Multiple Deprivation (SIMD), with a score of 1 indicating a “most deprived” area, and a score of 5 indicating a “least deprived” area [36].

Ethical approval was not deemed necessary due to the use of secondary data in this study. Data were fully anonymised and used in accordance with the Declaration of Helsinki [37]. The entire derived dataset was deidentified before being analysed, and all data are presented in aggregated form. This is standard procedure in accordance with Caldicott principles, and Caldicott approval was in place for the data used in this study (Caldicott Application 2176; 3 August 2021). 

Data analysis was performed using SPSS version 24 (IBM Corp, Armonk, NY, USA). Continuous variables are reported as the mean ± standard deviation (SD), while categorical variables are reported as the number/total and percentage (%). To separate parametric from nonparametric variables, normality tests using the Kolmogorov–Smirnov test were performed. For continuous variables, analysis of variance or the Kruskall–Wallis test was used. Chi-square tests, Fisher’s exact test, and linear and linear associations were used to compare categorical variables. Pearson’s correlation was used to assess the strength of association between variables of interest. Holm correction was used to correct for multiple comparisons. Results were considered significant for corrected *p* ≤ 0.05, and all tests were two-tailed.

## 5. Conclusions

In summary, we demonstrated several significant differences between patients presenting to a tertiary sleep disorders centre in adulthood whose NREM parasomnia commenced in childhood or adolescence and continued, compared to those whose NREM parasomnia commenced in adulthood for the first time. A family history of NREM disorders was much more likely to be present in the younger-onset groups, as was a history of atopy and concurrent traumatic life experiences. Underlying psychiatric comorbidities were more prevalent in adult-onset parasomnias. Further work on phenotyping NREM parasomnias according to age of onset and age of offset should continue, incorporating both medical and psychological comorbidities, to adequately inform controlled treatment trials in the future which are sorely lacking. 

## Figures and Tables

**Table 1:** Baseline characteristics of patients with NREM parasomnias split by age of onset (significance: *p* at 0.05/3 = 0.017).

		Childhood Onset (*N* = 156/307, 50.8%)	Adolescence Onset (*N* = 25/307, 8.1%)	Adult Onset (*N* = 126/307, 41.1%)	*p*-Value
Age at presentation (years) (*N*)		31.1 ± 9.9 (*N* = 151)	29.5 ± 9.0 (*N* = 25)	39.5 ± 15.0 (*N* = 120)	<0.001
Gender (*N*/T, %)	Male	65/156 (41.7%)	6/25 (24.0%)	62/126 (49.2%)	0.06
	Female	91/156 (58.3%)	19/25 (76.0%)	64/126 (50.8%)	
SIMD deprivation score (*n*/5) (*N*)		3.1 ± 1.4 (*N* = 155)	3.1 ± 1.3 (*N* = 24)	2.8 ± 1.3 (*N* = 121)	0.21
BMI (kg/m^2^) (*N*)		27.1 ± 6.2 (*N* = 137)	27.1 ± 4.9 (*N* = 24)	28.4 ± 6.6 (*N* = 102)	0.26
ESS (*n*/24) (*N*)		8.5 ± 5.3 (*N* = 135)	8.0 ± 3.7 (*N* = 23)	9.1 ± 5.9 (*N* = 108)	0.59
Subjective hours of sleep per night (h) (*N*)		6.3 ± 2.0 (*N* = 54)	5.9 ± 1.7 (*N* = 14)	5.8 ± 1.7 (*N* = 43)	0.46
Current smoker (*N*/T, %)		32/156 (20.5%)	3/25 (12.0%)	37/126 (29.4%)	0.08
Daily caffeine consumption (*N*/T, %)		71/156 (45.5%)	7/25 (28.0%)	43/126 (34.1%)	0.07
Alcohol consumption (*N*/T, %)	None	62/156 (39.7%)	12/25 (48.0%)	69/126 (54.8%)	0.17
	Occasionally	78/156 (50.0%)	11/25 (44.0%)	48/126 (38.1%)	
	Regularly	16/156 (10.3%)	2/25 (8.0%)	9/126 (7.0%)	
Marital status (*N*/T, %)	Single	78/146 (53.4%)	15/25 (60.0%)	40/104 (38.5%)	0.34
	Married	56/146 (38.4%)	9/25 (36.0%)	52/104 (50.0%)	
	Widower	1/146 (0.7%)	0/25 (0.0%)	2/104 (1.9%)	
	Divorced	8/146 (5.5%)	1/25 (4.0%)	9/104 (8.7%)	
	Separated	3/146 (2.1%)	0/25 (0.0%)	1/104 (1.0%)	
Shift work (*N*/T, %)		20/156 (12.8%)	6/25 (24.0%)	23/126 (18.3%)	0.22
Unrefreshed by sleep (*N*/T, %)		80/156 (51.3%)	11/25 (44.0%)	55/126 (43.7%)	0.41

Abbreviations: sig. = significance, *N* = number, T = total, SIMD = Scottish Index of Multiple Deprivation, BMI = body mass index, ESS = Epworth Sleepiness Scale.

**Table 2:** Parasomnia related characteristics in patients with NREM parasomnias split by age of onset (*p* at 0.05/3 = 0.017).

		Childhood Onset	Adolescence Onset	Adult Onset	*p*-Value
Family history (*N*/T, %)		56/156 (35.9%)	12/25 (48.0%)	27/126 (21.4%)	0.005
Occurrence in early sleep (*N*/T, %)		73/156 (46.8%)	6/25 (24.0%)	31/126 (24.6%)	0.001
Number of behaviours (*N*/T, %)	Single	26/149 (17.4%)	1/25 (4.0%)	38/118 (32.2%)	0.001
	Multiple	123/149 (82.6%)	24/25 (96.0%)	80/118 (67.8%)	
Duration of disorder (*N*/T, %)	<1 year	0/147 (0.0%)	2/24 (8.3%)	29/115 (25.2%)	<0.001
	1–5 years	2/147 (1.4%)	3/24 (12.5%)	38/115 (33.0%)	
	5–10 years	12/147 (8.2%)	6/24 (25.0%)	28/115 (24.4%)	
	>10 years	133/147 (90.5%)	13/24 (54.2%)	20/115 (17.4%)	
Frequency of behaviours (*N*/T, %)	<1/month	12/138 (8.7%)	2/23 (8.7%)	16/97 (16.5%)	0.41
	1/month–1/week	36/138 (26.1%)	6/23 (26.1%)	27/97 (27.8%)	
	>1/week	90/138 (65.2%)	15/23 (65.2%)	54/97 (55.7%)	
More distress to (*N*/T, %)	Myself	65/134 (48.5%)	13/19 (68.4%)	55/105 (52.4%)	0.49
	My partner	14/134 (10.5%)	2/19 (10.5%)	13/105 (12.4%)	
	Both	55/134 (41.0%)	4/19 (21.1%)	37/105 (35.2%)	
Amnesia for behaviour (*N*/T, %)	Aware	3/125 (2.4%)	0/21 (0.0%)	7/101 (6.9%)	<0.001
	Partial amnesia	33/125 (26.4%)	14/21 (66.7%)	19/101 (18.8%)	
	Full amnesia	89/125 (71.2%)	7/21 (33.3%)	75/101 (74.3%)	
Epilepsy-related (*N*/T, %)		1/156 (0.6%)	0/25 (0.0%)	1/126 (0.8%)	1.00

Abbreviations: *N* = number, T = total.

**Table 3:** NREM parasomnia subtype and other sleep disorders split by age of onset (*p* at 0.05/3 = 0.017).

		Childhood Onset	Adolescence Onset	Adult Onset	*p*-Value
Sleepwalking (*N*/T, %)	Simple	66/156 (42.3%)	13/25 (52.0%)	34/126 (27.0%)	<0.001
	Complex *	64/156 (41.0%)	9/25 (36.0%)	45/126 (35.7%)	
Night terrors (*N*/T, %)		90/156 (57.7%)	18/25 (72.0%)	42/126 (33.3%)	<0.001
SRED (*N*/T, %)		12/156 (7.7%)	1/25 (4.0%)	15/126 (11.9%)	0.40
SBS (*N*/T, %)		17/156 (10.9%)	2/25 (8.0%)	31/126 (24.6%)	0.005
Sleep-talking (*N*/T, %)		88/156 (56.4%)	12/25 (48.0%)	49/126 (38.9%)	0.014
Dream mentation (*N*/T, %)		62/156 (39.7%)	12/25 (48.0%)	25/126/ (19.8%)	<0.001
Violence in sleep (*N*/T, %)	To self	14/156 (9.0%)	5/25 (20.0%)	11/126 (8.7%)	0.22
	To others	30/156 (19.2%)	6/25 (24.0%)	16/126 (12.7%)	
	To both	9/156 (5.8%)	2/25 (8.0%)	8/126 (6.4%)	
Other sleep disorders (*N*/T, %)	Insomnia	6/156 (3.9%)	0/25 (0.0%)	4/126 (3.2%)	0.046
	Bruxism	4/156 (2.6%)	2/25 (8.0%)	7/126 (5.6%)	
	OSA	14/156 (9.0%)	1/25 (4.0%)	21/126 (16.7%)	
	RLS	3/156 (1.9%)	0/25 (0.0%)	2/126 (1.6%)	
	Sleep paralysis	15/156 (9.6%)	4/25 (16.0%)	11/126 (8.7%)	

Abbreviations: *N* = number, T = total, SRED = sleep-related eating disorder, SBS = sexualised behaviour in sleep, OSA = obstructive sleep apnoea, RLS = restless legs syndrome. * Interaction with the environment and associated with additional behaviours, e.g., speaking, screaming, and semi-purposeful movement [20].

**Table 4:** vPSG characteristics of patients with NREM parasomnia split by age of onset (*p* at 0.05/3 = 0.017).

	Childhood Onset	Adolescence Onset	Adult Onset	*p*-Value
Sleep onset latency (min) (*N*)	26.0 ± 19.9 (*N* = 112)	20.7 ± 19.2 (*N* = 18)	30.1 ± 46.1 (*N* = 84)	0.46
Wake time after sleep onset (min) (*N*)	77.8 ± 52.9 (*N* = 111)	80.3 ± 70.3 (*N* = 18)	88.9 ± 72.6 (*N* = 84)	0.47
Total sleep (min) (*N*)	355.3 ± 66.2 (*N* = 112)	360.7 ± 87.5 (*N* = 18)	335.6 ± 102.1 (*N* = 84)	0.22
Sleep efficiency (%) (*N*)	77.1 ± 12.9 (*N* = 112)	78.0 ± 18.6 (*N* = 18)	74.5 ± 20.9 (*N* = 84)	0.53
Total number of awakenings (*n*) (*N*)	31.0 ± 14.6 (*N* = 112)	32.8 ± 11.9 (*N* = 18)	36.9 ± 38.5 (*N* = 84)	0.31
Periodic leg movements per hour (*n*) (*N*)	3.5 ± 9.9 (*N* = 112)	2.5 ± 5.2 (*N* = 18)	6.7 ± 17.9 (*N* = 83)	0.20
Stage REM latency (min) (*N*)	136.2 ± 85.8 (*N* = 109)	103.7 ± 40.6 (*N* = 17)	108.5 ± 73.1 (*N* = 78)	0.04
AHI (*n*) (*N*)	10.2 ± 11.7 (*N* = 112)	12.1 ± 15.8 (*N* = 18)	19.3 ± 26.9 (*N* = 85)	0.005
Stage REM (%) (*N*)	17.6 ± 7.5 (*N* = 111)	18.3 ± 7.4 (*N* = 18)	16.1 ± 12.2 (*N* = 83)	0.48
Stage N1 (%) (*N*)	3.6 ± 4.3 (*N* = 111)	3.0 ± 6.1 (*N* = 18)	6.3 ± 13.2 (*N* = 83)	0.09
Stage N2 (%) (*N*)	57.6 ± 13.1 (*N* = 111)	61.7 ± 9.3 (*N* = 18)	56.0 ± 17.8 (*N* = 83)	0.32
Stage N3 (%) (*N*)	13.4 ± 11.9 (*N* = 111)	6.3 ± 7.8 (*N* = 18)	10.2 ± 13.5 (*N* = 83)	0.03
Presence of behaviour-related arousal from N3 stage	30 (*N* = 72)	1 (*N* = 3)	22 (*N* = 40)	0.36

Continuous variables are presented as the mean ± standard deviation. Abbreviations: vPSG = video polysomnography, *N* = number, AHI = apnoea–hypopnoea index, REM = rapid eye movement.

**Table 5:** Prevalence of atopy and asthma in patients with NREM parasomnia split by age of onset (*p* at 0.05/3 = 0.017).

	Childhood Onset	Adolescence Onset	Adult Onset	*p*-Value
Asthma (*N*/T, %)	21/156 (13.5%)	5/25 (20.0%)	17/126 (13.5)	0.61
Atopy (*N*/T, %)	26/156 (16.7%)	0/25 (0.0%)	6/126 (4.8%)	0.001

Abbreviations: *N* = number, T = total.

**Table 6:** Prevalence of psychiatric comorbidities and traumatic experiences in patients with NREM parasomnia split by age of onset (*p* at 0.05/3 = 0.017).

		Childhood Onset	Adolescence Onset	Adult Onset	*p*-Value
*Psychiatric comorbidity (N/T, %)*					
Any psychiatric comorbidity		58/156 (37.2%)	12/25 (48.0%)	69/126 (54.8%)	0.012
Depression		57/156 (36.5%)	15/25 (60.0%)	55/126 (43.7%)	0.07
Anxiety		57/156 (36.5%)	14/25 (56.0%)	55/126 (43.7%)	0.14
Self-harm		3/156 (1.9%)	1/25 (4.0%)	4/126 (3.2%)	0.63
Suicide attempt/ideation		11/156 (7.1%)	0/25 (0.0%)	11/126 (8.7%)	0.37
Chronic pain		8/156 (5.1%)	2/25 (8.0%)	9/126 (7.1%)	0.71
Chronic fatigue		1/156 (0.6%)	0/25 (0.0%)	2/126 (1.6%)	0.68
Traumatic experience (N/T, %) *					
Abortion		6/156 (3.9%)	0/25 (0.0%)	4/126 (3.2%)	1.00
Miscarriage		2/156 (1.3%)	0/25 (0.0%)	3/126 (2.4%)	0.78
Victim of assault		15/156 (9.6%)	2/25 (8.0%)	9/126 (7.1%)	0.78
Army or police officer		7/156 (4.5%)	0/25 (0.0%)	5/126 (4.0%)	0.82
PTSD		9/156 (5.8%)	3/25 (12.0%)	7/126 (5.6%)	0.48
Trauma *		43/156 (27.6%)	9/25 (36.0%)	39/126 (31.0%)	0.63
Trauma age	Childhood	37/47 (78.7%)	11/11 (100.0%)	17/42 (40.5%)	<0.001
	Adult	10/47 (21.3%)	0/11 (0.0%)	25/42 (59.5%)	
Trauma severity	Mild	12/49 (24.5%)	2/11 (18.2%)	7/42 (16.7%)	0.69
	Severe	37/49 (75.5%)	9/11 (81.8%)	35/42 (83.3%)	

Abbreviations: *N* = number, T = total, PTSD = post-traumatic stress disorder. * Trauma was defined using the ICD-10 trauma definition: *a ‘delayed or protracted response to a stressful event or situation (of either brief or long duration) of an exceptionally threatening or catastrophic nature, which is likely to cause pervasive distress in almost anyone’* [21]. For comparative purposes, the DSM-5 criteria (*‘actual or threatened death, serious injury, or sexual violence’*) were also used [22].

**Table 7:** Type of treatment for patients with NREM parasomnia split by age of onset (*p* at 0.05/3 = 0.017).

	Childhood Onset	Adolescence Onset	Adult Onset	*p*-Value
CBT (*N*/T, %)	13/156 (8.3%)	1/25 (4.0%)	8/126 (6.4%)	0.74
Counselling (*N*/T, %)	19/156 (12.2%)	7/25 (28.0%)	10/126 (7.9%)	0.02
Neuropsychological review (*N*/T, %)	8/156 (5.1%)	0/25 (0.0%)	6/126 (4.8%)	0.77
Psychiatrist (*N*/T, %)	21/156 (13.5%)	3/25 (12.0%)	12/126 (9.5%)	0.61
Hypnosis (*N*/T, %)	3/156 (1.9%)	1/25 (4.0%)	1/126 (0.8%)	0.36
Medication (*N*/T, %)	122/156 (78.2%)	21/25 (84.0%)	92/126 (73.0%)	0.39

Abbreviations: CBT = cognitive behavioural therapy, *N* = number, T = total.

**Table 8:** Efficacy of de novo pharmaceutical treatment in patients with NREM parasomnia split by age of onset (*p* at 0.05/3 = 0.017).

	Childhood Onset	Adolescence Onset	Adult Onset	*p*-Value
Amitriptyline (*N*/T, %) (10–100 mg)	26/156 (16.7%)	2/25 (8.0%)	8/126 (6.4%)	0.02
Effective (*N*/T, %)	7/26 (26.9%)	1/2 (50.0%)	1/8 (12.5%)	0.57
Lack of effect (*N*/T, %)	10/26 (38.5%)	0/2 (0.0%)	3/8 (37.5%)	
Sertraline * (*N*/T, %) (25–150 mg)	22/156 (14.1%)	5/25 (20.0%)	10/126 (7.9%)	0.11
Effective (*N*/T, %)	13/22 (59.1%)	0/5 (0.0%)	7/10 (70.0%)	0.05
Lack of effect (*N*/T, %)	0/22 (0.0%)	1/5 (20.0%)	0/10 (0.0%)	
Melatonin (*N*/T, %) (1–4 mg)	21/156 (13.5%)	5/25 (20.0%)	12/126 (9.5%)	0.25
Effective (*N*/T, %)	11/21 (52.4%)	3/5 (60.0%)	3/12 (25.0%)	0.12
Lack of effect (*N*/T, %)	5/21 (23.8%)	0/5 (0.0%)	6/12 (50.0%)	
Clonazepam (*N*/T, %) (0.25–2 mg)	86/156 (55.1%)	11/25 (44.0%)	52/126 (41.3%)	0.06
Effective (*N*/T, %)	42/86 (48.8%)	6/11 (54.5%)	25/52 (48.1%)	0.17
Lack of effect (*N*/T, %)	7/86 (8.1%)	3/11 (27.3%)	10/52 (19.2%)	
Zopiclone (*N*/T, %) (3.75–7.5 mg)	17/156 (10.9%)	6/25 (24.0%)	15/126 (11.9%)	0.20
Effective (*N*/T, %)	13/17 (76.5%)	1/6 (16.7%)	4/15 (26.7%)	0.43
Lack of effect (*N*/T, %)	2/17 (11.8%)	0/6 (0.0%)	3/15 (20.0%)	

Abbreviations: *N* = number, T = total. * Prescribed as a morning dose, primarily in the context of an elicited history of anxiety if not already on an anxiolytic drug. All other medications prescribed at night (or just prior to the major sleep period). Dose range is reported.

## Data Availability

Data are available on reasonable request to the corresponding author.

## Abbreviations

AHI	apnoea–hypopnoea index
BMI	body mass index
CBT	cognitive behavioural therapy
CNS	central nervous system
DSM-5	Diagnostic and Statistical Manual of Mental Disorders, Fifth Edition
ESS	Epworth Sleepiness Scale
HLA	human leukocyte antigen
ICSD-3	International Classification of Sleep Disorders (third edition)
NREM	non-rapid-eye-movement
OSA	obstructive sleep apnoea
REM	rapid eye movement
RLS	restless legs syndrome
sd	standard deviation
SBS	sexualised behaviour in sleep
SES	socioeconomic status
SIMD	Scottish Index of Multiple Deprivation
SRED	sleep-related eating disorder
SSRI	selective serotonin reuptake inhibitors
SWS	slow-wave sleep
vPSG	video polysomnography
